# Decoding dose descriptors for computed tomography

**DOI:** 10.1590/0100-3984.2023.0116

**Published:** 2024-04-15

**Authors:** Mannudeep K. Kalra, Lina Karout, Felipe de Moura Kiipper, Mônica Oliveira Bernardo

**Affiliations:** 1 Massachusetts General Hospital, Harvard Medical School, Boston, MA, USA; 2 Hospital Sírio-Libanês, São Paulo, SP, Brazil; 3 Hospital Dr. Miguel Soeiro, Pontifícia Universidade Católica de São Paulo (PUC-SP), Sorocaba, SP, Brazil

## INTRODUCTION

The Latin Safe initiative and the Massachusetts General Hospital Webster Center for Quality and Safety present a series of News in Radiology articles encompassing different aspects of radiation dose optimization in diagnostic imaging. Each article will present practical aspects that can help radiologists, technologists, and medical physicists understand and apply those concepts to improve patient safety and quality. This first article explains radiation dose descriptors for computed tomography (CT).

## RADIATION DOSE DESCRIPTORS

**Figure 1:**
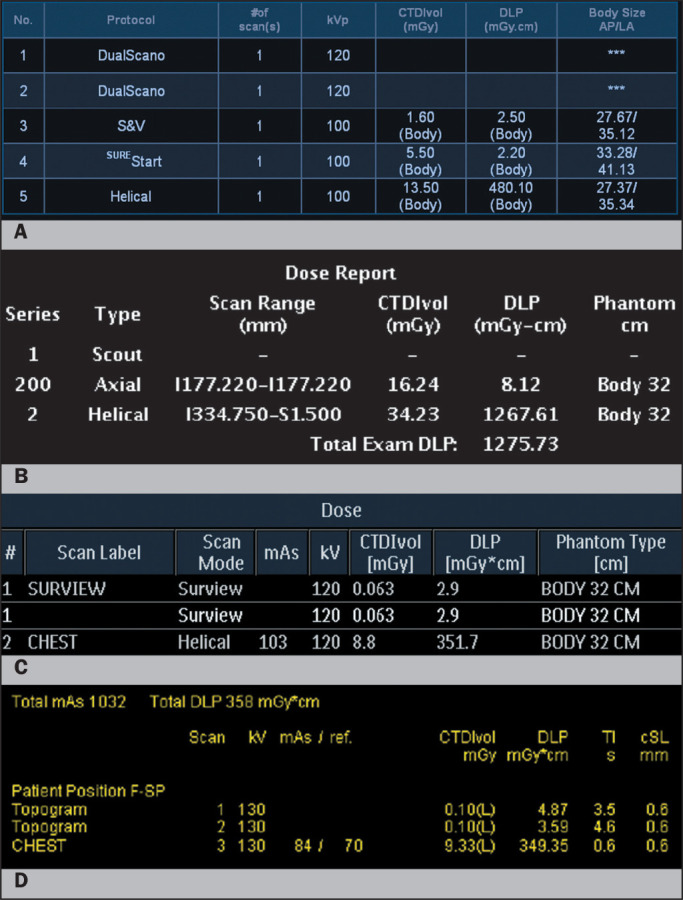
Dose information pages from four major CT vendors (A: Canon; B: GE; C: Philips; D: Siemens). Despite their differences, each vendor provides series-specific CTDIvol and DLP, as well as the corresponding phantom size, which enables users to compare radiation doses across different CT protocols and scanners. AP, anterior-posterior; LA, lateral; TI, (tube rotation) time; cSL, slice collimation; F-SP, feet first-supine.

Regulations require all CT scanners to display scanner output values^(1)^, including the volume CT dose index (CTDIvol, in mGy) and dose length product (DLP, in mGy.cm)-Figure 1. The CTDIvol represents the average radiation dose estimated by using polymethyl methacrylate phantoms of either 16 cm or 32 cm (typically for head and body CT, respectively). The DLP, defined as the total dose over the entire length of a scan, is the product of the CTDIvol and scan length.

The size-specific dose estimates derived from multiplying CTDIvol by a conversion factor based on patient size (measured diameters) compensate for the variation in patient sizes^(2)^. Diagnostic reference levels (DRLs) represent 75th-percentile radiation dose indices (CTDIvol and DLP) for a specific body region, examination type, or clinical indication, obtained from a survey of radiation doses at the local, regional, national, or international level^(3)^. The effective dose, a term coined by the International Commission of Radiation Protection, describes the relative whole-body radiation dose (in mSv), which is estimated by summing the individual organ doses or, more crudely, by multiplying the DLP by the conversion coefficients specific to the age of the patient and the body segment being imaged. Calculation of the effective dose allows radiation risks to be evaluated at the population level.

### Limitations

The CTDIvol and DLP represent absorbed doses in phantoms or the scanner output dose, rather than patient doses. These doses should not be used to estimate radiation risks associated with CT scanning. The DRLs should not be used for optimizing radiation doses on a per-patient basis, because they do not account for variations in patient size or scanner-specific attributes.

### Practical applications

Despite their limitations, CT radiation dose descriptors are powerful tools in radiation dose optimization. Because these descriptors are measured in a standardized manner, they allow dose comparison across all scanners and CT protocols. Given that they are displayed during the planning of the examination and before the actual scanning, users can modify scan factors to adjust the doses prior to scanning. In addition, the dose descriptors can be obtained either from a dose information page or from a structured Digital Imaging and Communications in Medicine report, either manually or automatically with radiation dose monitoring software. Dose monitoring allows institutions to compare their doses with those of other institutions or against internal target dose values.

Radiation doses are linked to image quality, and the specific clinical indication or motive should dictate the quality of the image. Therefore, the monitoring of radiation doses must involve documentation and analysis of the clinical indications or reasons for scanning^(4,5)^. With the technological revolution proceeding at an exponential pace, most scanners can automatically adapt radiation doses and maintain quality for different body parts and patient sizes. However, such automation fails if users do not make manual adjustments based on the clinical indications. It is imperative that all interpreting physicians and CT technologists know and understand the typical local values for CTDIvol and DLP at their institutions, as well as how those values compare with the regional, national, and international DRLs (Tables 1 and 2).

**Table 1:** Clinical indication-based DRLs in Brazil from a multicenter effort led by the Brazilian College of Radiology and Diagnostic Imaging. Although the DRLs for head CT are well below those employed in the United States and Europe, the DRLs for chest and abdomen CT suggest a need for protocol optimization and radiation dose reduction.

Body region	Clinical indication	Descriptor			
		CTDIvol (mGy)		DLP (mGy.cm)	
		AD_C|_	DRL_CI_	AD_C|_	DRL_CI_
Head	Head trauma	26	37	601	769
	Headache	29	45	616	955
	Stroke	30	53	617	998
	Head CTA	19	26	633	1,181
Paranasal sinus	Sinusitis	15	19	228	353
Cervical spine	Trauma	16	23	394	547
Chest	COVID-19	8	12	320	454
	Lung cancer	6	10	249	419
	Pneumonia	8	10	285	379
	Pulmonary embolism	11	15	376	582
Abdomen	Appendicitis	9	12	443	632
	Kidney stones	10	13	535	717
Chest and abdomen	Cancer	8	13	628	1,345

AD_CI_, achievable dose (based on the) clinical indication; DRL_CI_, DRL (based on the) clinical indication CTA, CT angiography.

**Table 2:** Summary of radiation doses for pediatric CT examinations in Latin America.

Examination	Age (years)	CTDIvol (mGy)		DLP (mGy.cm)	
		DRL (AD)	Range	DRL (AD)	Range
Unenhanced	0-< 1	27 (20)	3-113	456 (302)	35-1508
head CT	l-< 2	30 (20)	5-113	535 (356)	104-2528
	2-< 6	44 (29)	8-70	813 (527)	120-1650
	6-18	52 (35)	9-77	949 (625)	82-3023
Unenhanced	0-< 1	4(2)	0.19-14.0	81 (39)	3-230
chest CT	l-<5	4(2)	0.2-24.0	96 (49)	4-810
	5-< 10	5(4)	0.3-28.0	176(120)	8-743
	10-< 15	8(5)	0.4-28.0	294 (172)	9-1828
	15-18	11 (6)	0.9-22.0	425 (276)	19-1168
Contrast-	0-< 1	5(2)	0.2-13.0	83 (42)	3-277
enhanced	l-<5	5(3)	0.2-39.0	110 (62)	6-916
chest CT	5-< 10	6(4)	0.3-28.0	167 (107)	9-785
	10-< 15	9(6)	0.5-47.0	293 (202)	9-2077
	15-18	12 (8)	0.3-35.0	412 (305)	13-1846
Contrast-	0-< 1	3(2)	0.2-42.0	130 (46)	3-386
enhanced	l-<5	4(3)	2-29	160 (103)	9-794
abdomen-pelvis CT	5-< 10	7(4)	0.6-46.0	260 (163)	17-1298
	10-< 15	12 (7)	1-80	418 (292)	39-3027
	15-18	13 (9)	2.5-78.0	529 (414)	97-2028

Adapted from AAPM^(2)^. AD, achievable dose.

## CONCLUSION

Dose descriptors for CT are a powerful ally in the quest for radiation dose optimization. Radiologists and technologists should understand the strengths and weaknesses of these descriptors.
